# Early Electrophysiological Basis of Experience-Associated Holistic Processing of Chinese Characters

**DOI:** 10.1371/journal.pone.0061221

**Published:** 2013-04-11

**Authors:** Hui Chen, Cindy M. Bukach, Alan C.-N. Wong

**Affiliations:** 1 Department of Psychology, The Chinese University of Hong Kong, Shatin, N.T., Hong Kong; 2 Department of Psychology, University of Richmond, Richmond, Virginia, United States of America; University of Leicester, United Kingdom

## Abstract

Recent studies have found holistic processing to be a marker of expertise for perception of words in alphabetic (e.g., English) and non-alphabetic (e.g., Chinese) writing systems, consistent with what has been found for faces and other objects of face-like expertise. It is unknown, however, whether holistic processing of words occurs in an early, perceptual stage as it does for faces. We examined how early holistic processing of Chinese characters emerges by recording the event-related potentials (ERPs) in an adaptation paradigm. Participants judged if the top parts of two sequentially presented characters were the same or different while ignoring the bottom part. An early potential (P1) at the posterior channels was smaller when the attended top parts were the same compared with when they are different, indicating an adaptation effect. Critically, for trials with identical top parts, P1 was larger when the irrelevant bottom parts were different, indicating a release of adaptation. This effect was present only when the two character parts were aligned but not misaligned, and only for characters but not for pseudocharacters. The finding of early sensitivity to all parts of a Chinese character suggests that Chinese characters are represented holistically at a perceptual level.

## Introduction

Recent years have seen an increasing interest in the study of perceptual expertise for various object categories. In order to reveal both the common principles and unique aspects of different types of perceptual expertise, research has begun to compare the task demand, behavioral phenomena, and brain activity patterns for expert processing in different domains.

One important comparison involves perceptual expertise with faces and words. Face perception has traditionally been regarded as highly distinct from word perception [1], [2]. This is supported by evidence from both patient studies [3] and neuroimaging studies [4], [5] that different brain regions are involved in processing faces and words. A common view is that the requirement of fine-detailed, subordinate-level discrimination in face perception results in the development of holistic processing for faces, while words are perceived in a part-based manner because of the larger emphasis on coarser, basic-level categorization [2], [6].

Studies of face-like expertise also provide evidence consistent with the above view. Holistic processing has been found repeatedly for faces, as well as after the acquisition of face-like expertise for categories like cars, fingerprints, chessboards, and even novel objects [6], [7], [8], [9], [10], [11].

Curiously, our recent findings suggest that holistic processing is also a marker of expertise for words across alphabetic and non-alphabetic languages, such as English words [12] and Chinese characters [13]. In Wong et al. [12], we used the composite paradigm typical for measuring one aspect of holistic face processing: the obligatory processing of all parts of an object. Larger holistic processing for 4-letter English words was found for experts with English (native readers) than intermediate experts (second-language readers). More importantly, only native readers showed larger holistic processing for words than pseudowords. This experience-associated holistic processing has also been found for two-component Chinese characters with either a left-right or top-bottom structure, with native Chinese readers showing larger holistic processing for characters with valid than invalid part combinations [13].

Although the same paradigm (composite paradigm) has been used to reveal holistic processing for faces and for words, this does not mean that the same mechanisms underlie the effects for the two domains. For faces, holistic processing has been tied to the formation a holistic representation at an early phase of visual processing [14], [15], [16]. In Jacques and Rossion [14], for example, observers matched the top halves of two sequentially presented faces while ignoring the bottom halves. An early event-related potential (ERP) component over the right occipitotemporal region at about 160 ms (N170) was found to be sensitive not only to the changes of the target top part but also to the changes of the distractor bottom part. This sensitivity to changes in the distractor part disappeared when the top and bottom parts were misaligned, indicating that the holistic nature of the N170 requires an intact facial configuration. Results like this, in combination with similar findings in the fusiform gyus in fMRI studies [17], [18], suggest that faces are represented in a holistic manner during perceptual processing.

It is unknown whether holistic processing for words has a perceptual locus similar to that for faces. The expertise-related holistic processing of words demonstrated in the studies described above [12], [13] can be a result of one of the following possibilities. One, visual representations of words may be holistic, similar to face representations. Two, words may be represented in a piece-meal manner during the earlier perceptual phase and only integrated into a whole at a later, linguistic level.

**Figure 1:**
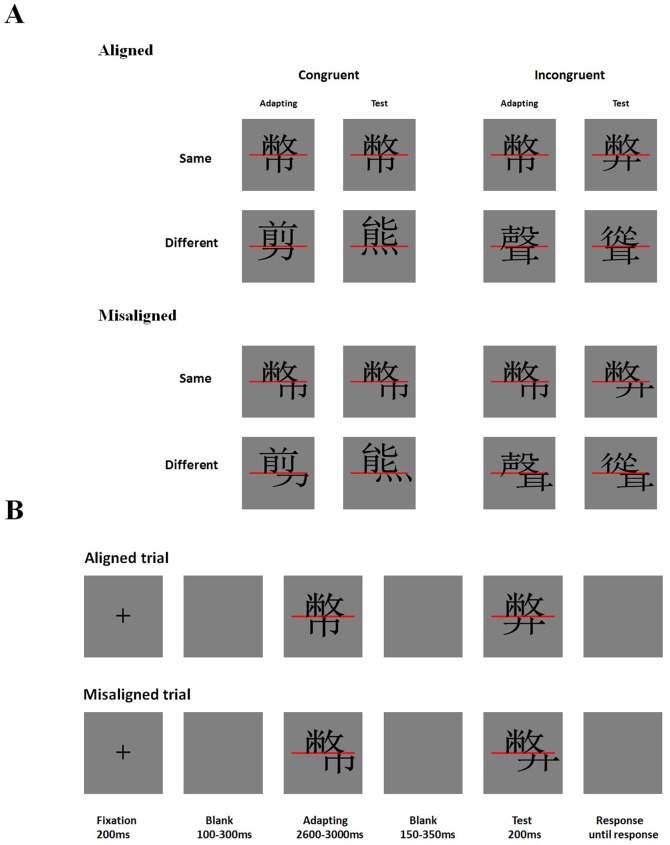
The stimuli and trial sequence in Experiment 1. (A) Examples of pairs of Chinese characters (adapting and test) for the different conditions. Participants judged if the top parts of the two characters were the same. (B) The trial sequence.

The current study was designed to test the first possibility that expertise with words results in the development of holistic processing at a perceptual level. Past studies have shown that early visual areas can be modified by perceptual learning [19], [20] and by specific learning experiences with letters and words [21], [22]. We recorded the ERPs of observers viewing characters in a composite paradigm. A sequential matching, full-design version of this paradigm was used [23], with Chinese characters having two parts arranged in a top-bottom configuration (Figure 1). On each trial, two characters were presented sequentially, and participants judged if the target parts (top) of the two characters were the same or different, while ignoring the distractor parts (bottom). Following previous findings [13], a congruency effect should be observed, i.e., performance should be better when the target and distractor parts agree (both tops and bottoms the same or both different) than when they disagree (one part the same and the other part different). Such a congruency effect represents interference from the distractor part, or, in other words, imperfect selective attention to the target part. Furthermore, this congruency effect should also be smaller when the overall configuration of a word is disrupted by misaligning the target and distractor parts. This alignment-dependent congruency effect is regarded as an indicator of holistic processing because it reflects one's obligatory attention to all parts of an object in a familiar configuration. Such an effect has not only been found for faces and words but also for other object categories of expertise like cars [8], [10] and novel objects [6], [24].

The composite task design also allows measurement of the amount of adaptation in early ERP components to repeated characters or character parts, which is useful for revealing the neural locus of holistic processing [14], [15], [16]. The congruent-same condition (Figure 1A), for example, involves presentation of two identical stimuli (tops and bottoms the same). This should result in adaptation or repetition suppression, i.e., reduced activation to the test stimulus compared with the study. The congruent-different (tops and bottoms different) and incongruent-different (tops different and bottoms same) conditions both involve changes in the target top part, and should thus lead to higher activation compared with the congruent-same condition, or a release of adaptation. The most critical condition is the incongruent-same condition (tops same and bottoms different). In this condition, the target top part remains the same while the distractor bottom part changes. If an observer can selectively attend to the top and ignore the bottom in a perfect manner, then we should expect as much adaptation as in the congruent-same condition. If, however, an observer cannot help but attend to all parts of a character, then the distractor changes should cause a release of adaptation, leading to larger activity for the incongruent-same condition than the congruent-same condition. We also investigated if the release of adaptation is modulated by misaligning the character parts. If expert character processing is similar to face processing in the requirement of an intact configuration, then disrupting the configuration of a character through misalignment should lead to suboptimal holistic processing and thus less or even no release of adaptation.

The current study targeted two ERP components associated with perceptual processing: P1 and N170. P1 is a positive component peaking at about 100 ms after stimulus onset around the lateral occipital channels. It has been regarded as reflecting perceptual processing in the extrastriate visual cortex [25]. Perceptual learning studies have shown that merely hours of laboratory training is enough to cause changes in early visual area activity [21]. It is conceivable that extensive reading experience can result in similar effects, given reading is an interactive activity involving both high-level linguistic and low-level visual processes. For example, Nazir [26] found differential visual field advantages for Hebrew and English words in Hebrew-English bilinguals. These results suggest that the regularity with which visual input falls in the retinal field during reading results in changes to early stages of visual analyses. fMRI studies have also shown selectively more responses to words than baseline objects controlled for low-level visual properties in the early visual areas including V1 to V4 [22]. Of note, P1 has been suggested to be associated with the holistic representation of a face, due to its sensitivity to the inversion of a face [27]. It was therefore one of the target components for which holistic processing of characters were examined. Adaptation to distractor part changes in the N1 would indicate an early locus of holistic processing, consistent with effects in the extra-striate cortex.

Another component of interest is the N170, a negative going component peaking at around 170 ms after stimulus onset at the posterior channels. The N170 in response to faces and other objects of expertise has been heavily studied [28], [29], [30], [31], [32], [33]. Source analyses as well as fMRI-ERP correlation studies have often tied the N170 for faces to activity in the fusiform gyrus and the lateral temporal cortex [34], [35], [36]. A left lateralized N170 has also been found to be selective for characters and words in one's familiar language across alphabetic and non-alphabetic writing systems [37], [38], [39], [40]. As discussed earlier, holistic face processing has been associated with N170 activity in previous studies using a similar paradigm as our current study [14], [15], [16]. We therefore identify N170 as the second target component in the current study.

We expected that ERP activity should be smaller when the target top parts of the two characters were the same than different, indicating adaptation to repeated presentation of the same stimuli. Critically, if holistic word processing has an early perceptual locus, we should observe a release of adaptation when the top target parts were the same but the bottom distractor parts were different. And if holistic processing requires an intact configuration of the characters, misaligning the top and bottom parts should attenuate such an adaptation. In Experiment 1 we tested valid Chinese characters, while in Experiment 2 pseudocharacters formed by illegal combinations of the same character parts were used.

## Experiment 1

### Methods

#### Ethics Statement

The procedures have been approved by the Survey and Behavioral Research Ethics Committee of the Chinese University of Hong Kong. Informed consent was obtained in written form from all participants.

#### Participants

Sixteen right-handed undergraduate students (4 females, mean age was 20.4 years) were recruited at the Chinese University of Hong Kong. They reported no history of neurological problems, all with normal or corrected-to-normal vision. They received monetary compensation for their participation.

#### Materials

The stimuli consisted of 120 pairs of traditional Chinese characters that have a top-bottom configuration, with 30 pairs in each of the four aligned conditions (Figure 1A). These aligned characters spanned 3.6° of visual angle vertically and 3.4° horizontally with a viewing distance of 60 cm. Top and bottom halves of aligned characters were separated by a red horizontal line. These characters were carefully selected so that there was no significant frequency difference between the characters in any two conditions [*p*s>.42]. In addition, 120 pairs of misaligned characters were generated by moving the bottom parts of aforementioned aligned characters slightly to the right (1.36° of visual angle). All the stimuli were in black, displayed against a light gray background in a 17-inch monitor using E-prime 1.1.

#### Procedure & Design

In each trial, two characters were presented sequentially in an aligned or misaligned format. At the start of each trial, a small white fixation cross was presented in the center of the display for 200 ms (Figure 1B). This was followed by a short blank interval of random duration (100 to 300 ms), after which the first character (adapting stimulus) was displayed for about 2800 ms (2600–3000 ms). The offset of the first character was followed by another blank interval of random duration (150 to 350 ms) and then the second character (test stimulus) was displayed for 200 ms. A blank response window then appeared and remained on the screen until participants responded. Participants were told to judge whether the top parts (above the red line) of the first and second stimuli were the same or not by pressing one of two response keys. After responding, an inter-trial-interval of about 1600 ms (1500 to 1700 ms) followed before the next trial began.

There were 90 trials for each of the 8 conditions in this 2×2×2 (alignment × congruency × correct response) factorial design, resulting in a total of 720 trials divided into 9 blocks. Trials of the 8 conditions were presented randomly within each block. Participants completed 16 practice trials before the actual experimental trials began.

#### Electrophysiological Recording and Analysis

EEG was recorded from 64 scalp sites using Ag/AgCl electrodes mounted in an elastic cap. All recordings were initially referenced to a vertex channel (CPZ), and re-referenced offline to the average of the whole head. Vertical electrooculogram (VEOG) was recorded with pairs of electrodes placed above and below the left eye, while the horizontal electrooculogram (HEOG) were recorded with pairs of electrodes placed beside the two eyes. Impedances were maintained below 5ΚΩ. The EEG and EOG were amplified by SynAmps using a 0.05–100 Hz bandpass filter and continuously sampled at 1000 Hz/channel for off-line analysis. The EEG was epoched into periods of 900 ms, from 100 ms before the onset of the test stimulus. The EEG and EOG were digitally filtered offline with a 30 Hz low-pass filter. Trials contaminated by amplifier clipping, bursts of electromyographic activity, or peak-to-peak deflection were excluded from the average by an automatic rejection algorithm with a threshold amplitude of ±75 µV.

Based on previous studies about holistic processing of faces [14], [28], the mean amplitudes of the P1 (maximal at approximately 120 ms in our experiment) for the time window of 100–140 ms and N170 (maximal at approximately 180 ms in our experiment) for the time window of 160–200 ms at four pairs of posterior sites (P7/8, PO7/8, CB1/2, O1/2) were analyzed. Alignment (aligned, misaligned), congruency (congruent, incongruent), and hemisphere (left, right) were taken as factors for repeated measures analyses of variance (ANOVA). Analysis of release of adaptation relied on trials with same responses only. The critical question is, within the same trials (where the target parts were the same), whether ERP potentials would have a larger amplitude for incongruent (distractor parts different) than congruent (distractor parts the same) trials due to release of adaptation, and whether it would occur for aligned trials but not misaligned trials.

### Results

#### Behavioral Data

**Figure 2:**
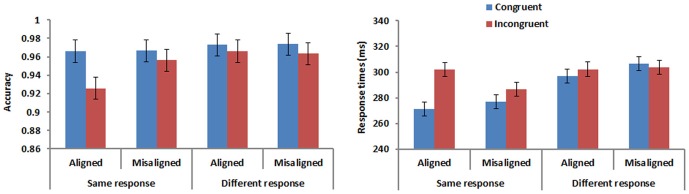
Accuracy and response times in Experiment 1. Error bars represent 95% confidence interval for the congruency factor.

The accuracy and mean correct response times (RTs) were shown in Figure 2. Congruency by alignment effects occurred in same trials only. Responses were more accurate and faster for congruent than incongruent trials, and this congruency effect occurred only for aligned but not misaligned trials. Accuracy and RTs were also submitted to repeated measures analyses of variance ANOVA with alignment (aligned, misaligned), congruency (congruent, incongruent), and response (same, different) as factors.

Accuracy. ANOVA performed on accuracy yielded a significant effect of congruency [*F*(1,15) = 11.230, *p*<.005, *η*_p_^2^ = .429], an interaction between congruency and alignment [*F*(1,15) = 6.275, *p*<.05, *η*_p_^2^ = .250], and a three-way interaction [*F*(1,15) = 5.482, *p*<.05, *η*_p_^2^ = .250].

To investigate these differences more thoroughly, we conducted two separate 2 (alignment) × 2 (congruency) ANOVAs for the same and different response conditions. The analysis for same response trials showed an effect of congruency [*F*(1,15) = 10.769, *p*<.01, *η*_p_^2^ = .417] and an interaction between congruency and alignment [*F*(1,15) = 16.440, *p*<.005, *η*_p_^2^ = .571]. As expected, responses in the congruent condition were more accurate than those in the incongruent condition and this difference was larger for aligned than misaligned trials, a pattern that is consistent with that found both in face and English word perception. Planned comparisons revealed that the difference between congruent and incongruent conditions was only significant in aligned trials [*t*(15) = 3.896, *p*<.005, *d* = .983], but not in misaligned trials [*t*(15) = 1. 656, *p*>.11]. The analysis for different response trials did not reveal any significant effects [all *p*s>.17].

Response time. Response times (based on correct responses) faster than 100 ms, slower than 2000 ms and greater than +/−3 standard deviations from the mean were removed. Consistent with accuracy, subjects responded faster in the congruent than incongruent condition [*F*(1,15) = 13.871, *p*<.005, *η*_p_^2^ = .480], and this congruency effect was larger for the aligned than misaligned trials [*F*(1,15) = 19.987, *p*<.001, *η*_p_^2^ = .571]. Planned comparisons revealed that the difference between congruent and incongruent conditions was only significant in aligned trials [*t*(15) = 5.217, *p*<.001, *d* = .246], but not in misaligned trials [*t*(15) = .959, *p*>.35].

For ease of comparison with the analysis of accuracy detailed above, we carried out two separate 2 (alignment) × 2 (congruency) ANOVAs on RTs for same and different response trials even though the 3-way interaction was not significant for RTs. There was a significant interaction between congruency and alignment in both same response [*F*(1,15) = 11.312, *p*<.005, *η*_p_^2^ = .430] and different response trials [*F*(1,15) = 6.950, *p*<.05, *η*_p_^2^ = .317].

#### ERP Data

**Figure 3:**
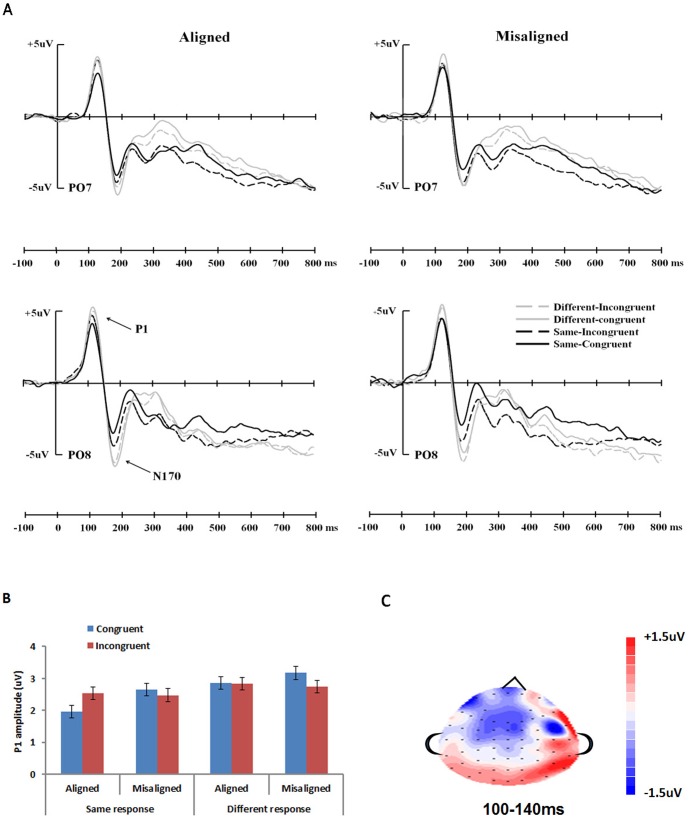
ERP results in experiment 1. (A) Grand average ERPs elicited by the test Chinese characters in different conditions at occipitotemporal electrodes PO7 and PO8. (B) Histograms of the amplitude of the P1 in the different conditions averaged over 4 pairs of electrodes (CB1/2, O1/2, PO7/PO8, P7/P8). Error bars represent 95% confidence interval for the congruency factor. (C) Topographical distributions of the difference in the release of adaptation effects (i.e., same-incongruent minus same congruent) between aligned and misaligned trials at 100–140 ms, which represent the range of P1 for different conditions. The red regions indicate the locations where the release of adaptation were greater for aligned than misaligned trials.

Grand average ERP waveforms elicited by the test stimuli in the various conditions are displayed in Figure 3A for two occipitotemporal electrodes (PO7/PO8). Both P1 and N170 showed a clear adaptation effect, with larger amplitudes for trials with both target and distractor parts changed (different-congruent condition) compared with repeated presentation of the same stimulus (same-congruent condition). When considering the same trials only, P1 showed a significantly larger amplitude for incongruent than congruent trials, indicating a release of adaptation, only when the character parts were aligned but not when they were misaligned (Figure 3B). The congruency effect for the N170, however, was present for both aligned and misaligned trials. There was not a clear hemisphere effect for either component.

ERP data collapsed across the 4 channels on each hemisphere were submitted to two analyses: (i) The same-congruent (target-same-distractor-same) and the different-congruent (target-different-distractor-different) conditions were compared with *t*-tests. Smaller activity for the former than latter condition would indicate an adaptation effect. (ii) Repeated measures analyses of variance (ANOVAs) with alignment (aligned, misaligned), congruency (congruent, incongruent), and hemisphere (left, right) as independent factors were analyzed separately for same and different trials. A larger emphasis was put on the same trials, since a larger activity for the same-incongruent (target-same-distractor-different) than same-congruent (target-same-distractor-same) condition would indicate a release of adaptation due to sensitivity to distractor changes. If this congruency effect occurs only for aligned trials (i.e., an alignment × congruency interaction), it would indicate that sensitivity to distractor part changes requires the presence of an intact character configuration. Only amplitude data were reported below since no significant effects were found involving latency (*p*s>.091).

P1 amplitude. First, the adaptation effect was examined by comparing the same-congruent (target-same-distractor-same) and the different-congruent (target-different-distractor-different) conditions. This was significant for both aligned [*t*(15) = 3.659, *p*<.005, *d* = .414] and misaligned conditions [*t*(15) = 2.179, *p*<.05, *d* = .261], indicating an adaptation effect due to stimulus repetition.

Next, for the same trials, an ANOVA showed a significant effect of alignment [*F*(1,15) = 5.839, *p*<.05, *η*_p_^2^ = .280] and a significant interaction between congruency and alignment [*F*(1,15) = 6.014, *p*<.05, *η*_p_^2^ = 286]. The interaction effect revealed that the P1 was larger in the incongruent than in the congruent condition, but only for the aligned trials [*t*(15) = 3.367, *p*<.005, *d* = .298] and not the misaligned trials [*t*(15) = .732, *p*>.47]. There was no significant main effect or interaction involving hemisphere. As shown in the topographical distribution (Figure 3C), the posterior channels of the two hemispheres both showed a release of adaptation effect that was larger for aligned than misaligned trials. Analysis for different response trials did not show any significant effects (all *p*s>.11).

N170 amplitude. The N170 also showed an adaptation effect, with a significantly larger amplitude for same-congruent than different-congruent condition for both aligned [*t*(15) = 3.760, *p*<.005, *d* = .579] and misaligned trials [*t*(15) = 3.796, *p*<.005, *d* = .507].

For the same trials, there were only significant main effects of alignment [*F*(1,15) = 5.277, *p*<.05, *η*_p_^2^ = .260] and congruency [*F*(1,15) = 16.846, *p*<.001, *η*_p_^2^ = .529]. We also examined the N170 amplitude after correcting for P1 amplitude and found a significant effect of congruency [*F*(1,15) = 37.312, *p*<.001, *η*_p_^2^ = .713]. Importantly, the alignment × congruency interaction and the alignment × congruency × hemisphere interactions were not significant [*p*s>.11].

For the different trials, there was an effect of alignment [*F*(1,15) = 4.620, *p*<.05, *η*_p_^2^ = .224], an alignment × congruency interaction [*F*(1,15) = 13.651, *p*<.005, *η*_p_^2^ = .460], and a hemisphere × congruency interaction [*F*(1,15) = 4.896, *p*<.05, *η*_p_^2^ = .234]. No significant effects were found, however, for the N170 amplitude after correcting for P1 amplitude [*p*s>.058].

## Experiment 2

In Experiment 1, we found that holistic processing of characters had an electrophysiological basis at the P1 component, starting at about 80 ms upon presentation. P1 amplitude was sensitive to changes in parts of a character that should not be attended to according to task instruction, therefore reflecting obligatory attention paid to all the parts of a character, or holistic processing. Importantly, this sensitivity to changes in all parts of a character occurred only when the parts form a valid configuration, and disappeared when the top and bottom parts were misaligned. In Experiment 2, we studied the effect of experience on such holistic processing, by examining if similar results would be found for pseudocharacters. It should be noted that the same character parts were used to form the pseudocharacters in Experiment 2 and the characters in Experiment 1, with the only difference in part combinations.

### Methods

**Figure 4:**
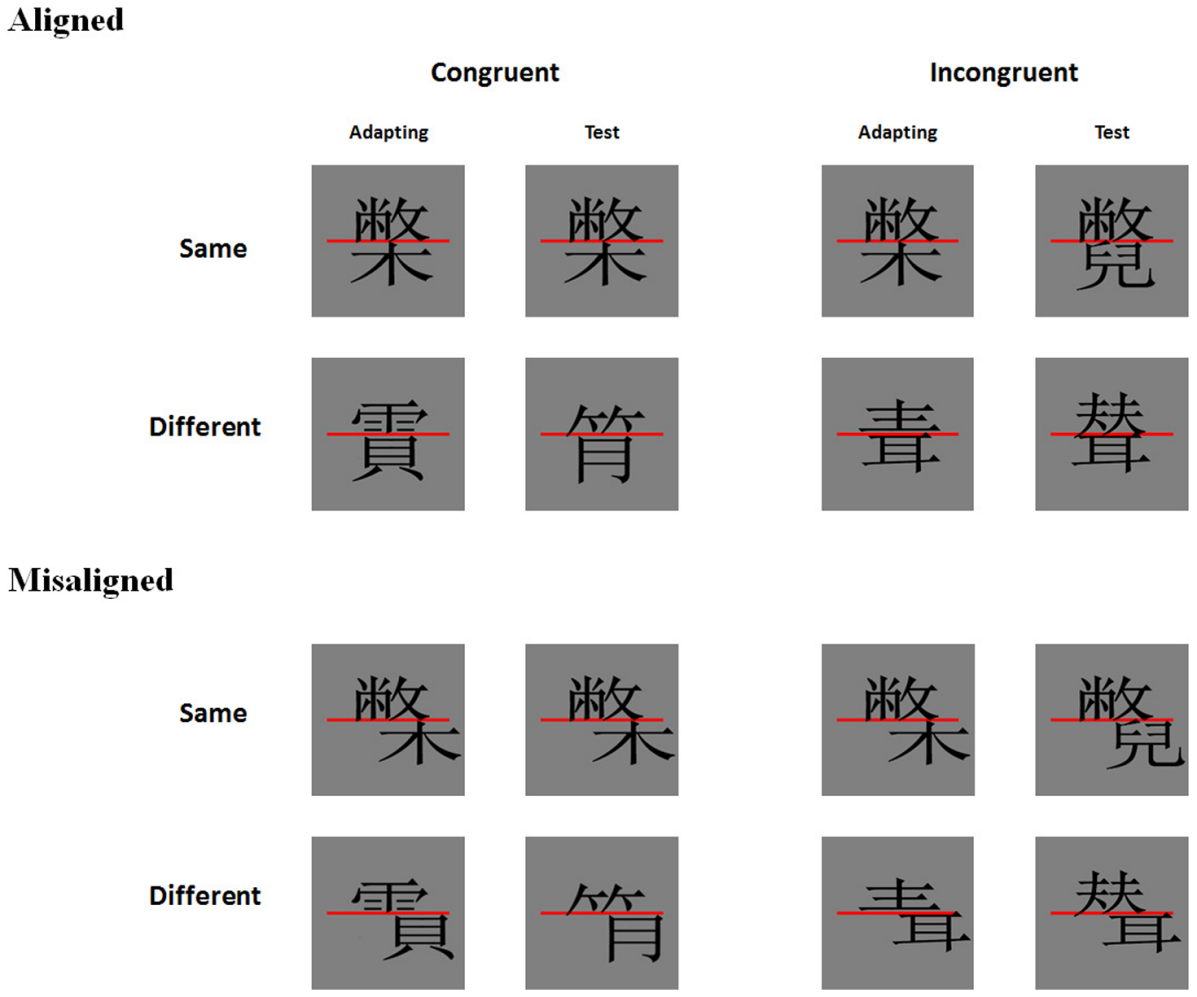
Examples of pairs of Chinese pseudocharacters (adapting and test) for the different conditions in Experiment 2. Participants judged if the top parts of the two characters were the same.

The materials, procedure, data collection and analysis methods used were identical to those of Experiment 1 with the following exceptions. First, a new group of sixteen right-handed students (7 females, mean age was 20.7 years) from the Chinese University of Hong Kong participated at this experiment. Second, the stimuli used in this experiment were Chinese pseudocharacters (see Figure 4), which were generated by randomly combining top and bottom parts from two different Chinese characters within the same condition from Experiment 1.

### Results

#### Behavioral Data

**Figure 5:**
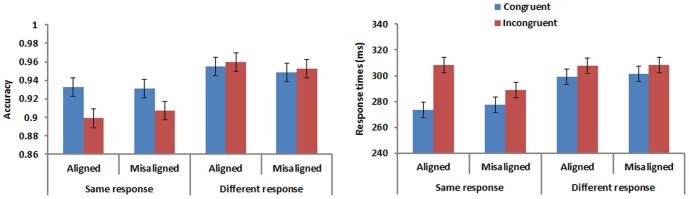
Accuracy and response times in Experiment 2. Error bars represent 95% confidence interval for the congruency factor.

Figure 5 presents the accuracy and mean correct RTs for Experiment 2. Differences occur mainly in the same trials, where responses were more accurate and faster for congruent than incongruent trials. The difference seemed larger when the two character parts were aligned than misaligned.

Accuracy. A three-way ANOVA on accuracy showed that subjects responded more accurately in the congruent than incongruent condition [*F*(1,15) = 5.388, *p*<.05, *η*_p_^2^ = .263]. This congruency effect was larger for same than different trials [*F*(1,15) = 8.657, *p*<.01, *η*_p_^2^ = .375], but similar for aligned and misaligned trials [*F*<1].

Separate analyses for same and different trials showed that, for same trials, there was a significant congruency effect [*F*(1,15) = 8.558, *p*<.01, *η*_p_^2^ = .368] and it did not interact with alignment [*F*<1]. For different trials, no effects were significant [*p*s>.13].

Response time. A three-way ANOVA conducted on RTs showed a response × alignment × congruency interaction [*F*(1,15) = 5.760, *p*<.05, *η*_p_^2^ = .277]. The two separate 2 (alignment) × 2 (congruency) ANOVAs for same and different trials revealed a congruency effect that depended on alignment only in the same response trials [*F*(1,15) = 13.520, *p*<.005, *η*_p_^2^ = .474]. For the different trials the congruency effect [*F*(1,15) = 9.595, *p*<.01, *η*_p_^2^ = .390] did not interact with alignment [*F*<1].

#### ERP Data

**Figure 6:**
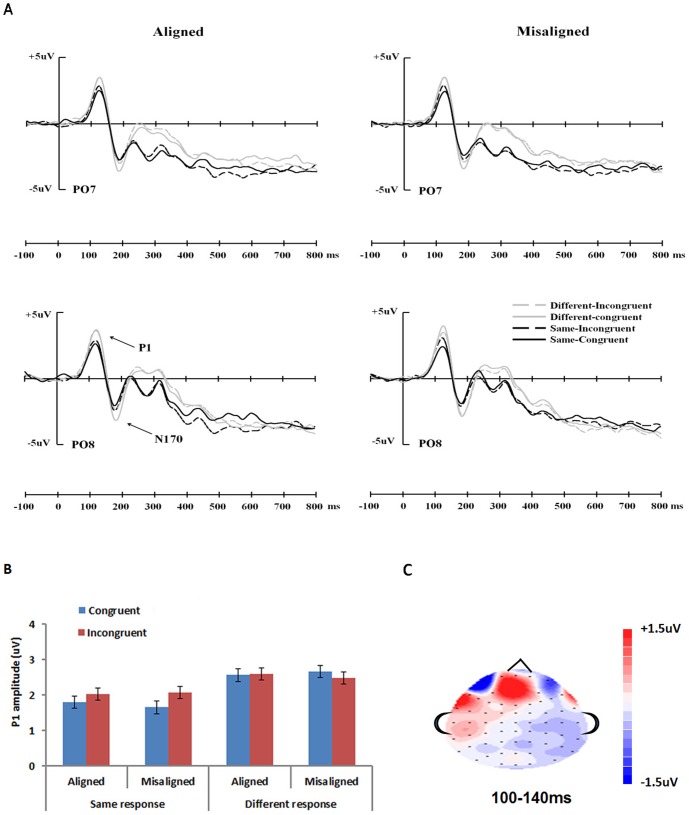
ERP results in experiment 2. (A) Grand average ERPs elicited by the test Chinese pseudocharacters in different conditions at occipitotemporal electrodes PO7 and PO8. (B) Histograms of the amplitude of the P1 in the different conditions averaged over 4 pairs of electrodes (CB1/2, O1/2, PO7/PO8, P7/P8). Error bars represent 95% confidence interval for the congruency factor. (C) Topographical distributions of the difference in the release of adaptation effects (i.e., same-incongruent minus same congruent) between aligned and misaligned trials at 100–140 ms, which represent the range of P1 for different conditions.

Figure 6A displays the grand average ERP waveforms elicited by the test stimuli in different conditions at two occipitotemporal electrodes (PO7/PO8). P1 and N170 displayed the typical adaptation effect, with a smaller amplitude to repeated presentation of the same stimulus (same-congruent condition) than presentation of two different stimuli (different-congruent condition). Concerning the same trials only, P1 showed a larger amplitude to incongruent than congruent same trials, indicating a release of adaptation (Figure 6B). However, this effect seemed unaffected by the misalignment of the top and bottom parts. The N170 did not show any systematic release of adaptation. As in Experiment 1, we first examined the adaptation effect by comparing same-congruent (target-same-distractor-same) and the different-congruent (target-different-distractor-different) conditions using a t-test. We then conducted repeated measures ANOVAs with alignment, congruency, and hemisphere as independent factors separately for same and different trials, with a larger emphasis on the same trials. Again, larger activity for the incongruent than congruent conditions for aligned trials only (an alignment × congruency interaction) would indicate a release of adaptation due to distractor part changes. As in experiment 1, there was no significant effect on latency (all *p*s>.15) and therefore only amplitude data were present as follows.

P1 amplitude. P1 revealed adaptation effects on both aligned [*t*(15) = 5.035, *p*<.001, *d* = .582] and misaligned trials [*t*(15) = 4.674, *p*<.001, *d* = .747], with a larger amplitude in different-congruent than same-congruent condition.

For same trials, a hemisphere × alignment × congruency ANOVA showed only a main effect of congruency [*F*(1,15) = 11.105, *p*<.005, *η*_p_^2^ = .425] and no other effects [*p*s>.27]. Although P1 has a slightly larger amplitude for incongruent than congruent trials, misalignment did not modulate this difference. Topographical maps showed a consistent lack of difference in the release of adaptation between aligned and misaligned trials in the posterior channels (Figure 6C). No effect was found for the different trials [*p*s>.074].

N170 amplitude. The adaptation effect (i.e., smaller N170 for same-congruent than different-congruent condition) was significant in both aligned [*t*(15) = 3.099, *p*<.01, *d* = .388] and misaligned conditions [*t*(15) = 2.545, *p*<.05, *d* = .355].

No significant effect was found for the same trials [*p*s>.13]. When correcting for the P1 amplitude, there were significant effects of alignment [*F*(1,15) = 4.575, *p*<.05, *η*_p_^2^ = .234] and congruency [*F*(1,15) = 11.316, *p*<.005, *η*_p_^2^ = .430], but their interaction as well as other effects were not significant [*p*s>.096]. For the different trials, there was a significant effect of congruency [*F*(1,15) = 8.501, *p*<.005, *η*_p_^2^ = .362], and it remained significant after correcting for P1 amplitude [*F*(1,15) = 7.124, *p*<.05, *η*_p_^2^ = .322]. No other effects were significant [*p*s>.096].

## General Discussion

While research findings have suggested that faces are represented holistically in the right hemisphere before 200 ms, the neural locus of holistic processing of words has not been examined. We studied Chinese characters as an example of words, and measured ERP responses in different conditions of a composite paradigm typically used for faces. Observers judged whether the tops of two sequentially presented characters were the same or not, ignoring the irrelevant bottom parts. The influence of the irrelevant part on one's judgment and ERP was used to indicate holistic processing of a character. As in previous adaptation studies, repeated presentation of the same character resulted in reduced activity compared with sequential presentation of two different characters (both tops and bottoms different, as in different-congruent trials). The critical finding in the current study was that changing the distractor part while keeping the target part the same (as in same-incongruent trials) led to a release of adaptation as early as around 80–120 ms after stimulus onset, as reflected in the P1 component.

While P1 reflects extrastriate processing and is susceptible to low-level visual differences [27], [41], low-level visual differences alone cannot provide a sufficient explanation for the current results. In Experiment 1, we found that the release of adaptation in P1 due to distractor changes occurred only when the character parts were aligned (but not when they were misaligned). This means that the release of adaptation found in the aligned condition cannot be caused merely by the low-level visual differences between the adapting and test stimuli. In addition, the P1 effects are likely associated with one's experience with the characters, as the P1 effect for pseudocharacters in Experiment 2 was much smaller and did not change with the alignment of the top and bottom parts.

An interesting characteristic of the current findings is that the release of adaptation happened at P1 but not at N170, while in previous studies selectively higher activity for Roman letters, English words, as well as Chinese characters than control stimuli have been found at N170 for observers familiar with the corresponding writing systems [38], [40]. Such a discrepancy between adaptation and selectivity may, however, be less surprising considering that adaptation and selectivity are not necessarily manifested by the same underlying neural mechanisms [42], [43]. Another potential reason is that adaptation can occasionally be more sensitive than selectivity measures in the study of form perception [44]. Consistent with this claim, a recent ERP study used a large number of trials per condition (700+) to boost its power and showed early selectivity for musical notes than symbols in musicians but not novices starting at 40–60 ms after stimulus onset [45]. Together with the fMRI finding of earlier specialization for words than visually controlled patterns in primary visual cortex [22], our data suggest an intriguing possibility that a more powerful design can find a neural locus of letter and word processing at a much earlier time frame than N170.

While the P1 effects found in the current study suggest an involvement of the extrastriate regions in the holistic representation of characters, one may argue that the effect could be caused by feedback from later, linguistic regions. Perhaps P1 may reflect extrastriate activity not only during the very first feed-forward processing sweep but also after the feedback from higher areas [46], [47], specifically from visual word form area [48] or areas responsible for phonological or semantic processing. One way to examine this possibility is to measure how higher-level factors modulate the magnitude of the release of adaptation. For example, would the effect be different when the top parts of the study and test characters are characters (with a good word form) themselves, compared with trials when the top parts are radicals? In our study, however, there were not sufficient trials for robust analyses for characters vs. radicals. The feedback possibility seems unlikely, however, for two reasons. First, we did not find any systematic release of adaptation dependent on alignment at late ERP components. Second, while feedback from left-lateralized linguistic processing is supposed to result in a left-lateralized P1 effect, our effect was bilateral.

Overall, our findings suggest that holistic processing (or the obligatory attention to all parts) of a character happens at an early, perceptual phase.
